# Cross-Cultural Adaptation and Psychometric Validation of the YFAS 2.0 for Assessing Food Addiction in the Mexican Adult Population

**DOI:** 10.3390/bs15081023

**Published:** 2025-07-28

**Authors:** Haydee Alejandra Martini-Blanquel, Indira Rocío Mendiola-Pastrana, Rubí Gisela Hernández-López, Daniela Guzmán-Covarrubias, Luisa Fernanda Romero-Henríquez, Carlos Alonso Rivero-López, Geovani López-Ortiz

**Affiliations:** 1 Unidad de Medicina Familiar Número 33, Instituto Mexicano del Seguro Social, Ciudad de México 02100, Mexico; haydee.martini@imss.gob.mx; 2 Hospital General de Zona con Unidad de Medicina Familiar Número 8, Instituto Mexicano del Seguro Social, Ciudad de México 01090, Mexico; indira.mendiola@imss.gob.mx; 3 Subdivisión de Medicina Familiar, Facultad de Medicina, Universidad Nacional Autónoma de México, Ciudad de México 04510, Mexico; mc21gucd7467@facmed.unam.mx (D.G.-C.); cr2025@fmposgrado.unam.mx (C.A.R.-L.); 4 Oficina de Análisis del Plan de Salud, Subgerencia Técnica de Plan de Salud, Gerencia de Administración del Plan de Salud, Banco de México, Ciudad de México 06000, Mexico; rhernandezl@banxico.org.mx; 5 Facultad de Filosofía y Letras, Universidad Nacional Autónoma de México, Ciudad de México 04510, Mexico; luisa.romeroh@aefcm.gob.mx

**Keywords:** food addiction, validation, psychometric properties, factor analysis, eating behavior, eating disorders

## Abstract

Food addiction is characterized by compulsive consumption and impaired control over highly palatable foods, with neurobiological mechanisms analogous to substance use disorders. The Yale Food Addiction Scale 2.0 (YFAS 2.0) is the most widely used instrument to assess these symptoms; however, its psychometric properties have not been validated in Mexican adults. This study aimed to perform the cross-cultural adaptation of the YFAS 2.0 and validate its psychometric properties for the identification of food addiction in the Mexican adult population. A cross-sectional study was conducted in 500 Mexican adults aged 20 years or older. Participants completed the cross-culturally adapted YFAS 2.0. Exploratory and hierarchical factor analyses were conducted. Reliability was assessed using Cronbach’s alpha and omega coefficients, and model fit was evaluated through global fit indices. The scale showed high internal consistency (α = 0.88; ωt = 0.87; ωh = 0.89). The Kaiser–Meyer–Olkin index was 0.815 and Bartlett’s test was significant (χ^2^ = 4367.88; df = 595; *p* < 0.001). Exploratory factor analysis supported a unidimensional structure, with the first factor explaining 21.3% of the total variance. In the hierarchical model, all items loaded substantially onto the general factor. Fit indices indicated excellent model fit (CFI = 0.99; TLI = 0.98; RMSEA = 0.001; RMR = 0.004). The YFAS 2.0 is a valid and reliable instrument for identifying food addiction symptoms in Mexican adults. It may be useful in clinical practice and research on eating disorders.

## 1. Introduction

In Mexico, the burden of noncommunicable chronic diseases has reached critical levels, posing substantial challenges to the national health system. Recent data from the National Health and Nutrition Survey (ENSANUT) indicate that more than 75% of adults are overweight or obese, 18.3% have type 2 diabetes mellitus (T2DM), 31.1% live with hypertension (HTN), and 30.4% have dyslipidemia (Barquera et al., 2024; Campos-Nonato et al., 2024; Escamilla-Nuñez et al., 2023; Rojas-Martínez et al., 2024). Despite this high prevalence, metabolic control remains limited: only 57.4% of patients with HTN and 34.4% of those with T2DM meet therapeutic goals (Basto-Abreu et al., 2024; Campos-Nonato et al., 2024). These figures highlight the need to identify behavioral factors that contribute to the persistence of these risk profiles.

In this context, the concept of food addiction has gained increasing attention in clinical and neurobiological research. Despite this growing interest, there is no consensus regarding its classification. It has been proposed that the phenomenon can be conceptualized using diagnostic criteria for substance-related disorders, as outlined in the DSM-5, although it has also been argued that it may align more closely with behavioral addiction models not based on substance use (Hauck et al., 2020). One of the most common descriptions defines food addiction as a loss of control over the consumption of highly palatable foods—typically rich in sugars, fats, and salt—despite negative consequences. This behavior is thought to activate brain reward pathways, particularly dopaminergic systems composed of neurons that release dopamine in response to pleasurable stimuli, in a manner similar to addictive substances such as alcohol and nicotine (Halbeisen et al., 2025; Li et al., 2024).

This perspective is supported by growing evidence linking food addiction to adverse metabolic outcomes. A systematic review estimated that nearly 20% of adults worldwide meet criteria for food addiction, with higher prevalence among individuals with obesity and clinical comorbidities (Pursey et al., 2014). In Mexico, recent analyses have highlighted the potential role of addictive eating patterns in sustaining high rates of obesity, diabetes, and related conditions, particularly within a food environment saturated by ultra-processed, low-cost, high-reward foods (Meléndez-Diego et al., 2022). A population-based study among Mexican university students reported a prevalence of 13.8% for probable food addiction and 8.1% for clinically significant cases (Munguía et al., 2022). These findings suggest that excessive food intake may not be solely volitional but may reflect compulsive consumption driven by altered neurobiological mechanisms, reinforcing the clinical relevance of food addiction as a target of assessment and intervention.

Standardized instruments identifying and quantifying food addiction symptoms are essential for epidemiological research and clinical practice. The Yale Food Addiction Scale (YFAS) has become one of the most widely used tools for this purpose (Gearhardt et al., 2009). Its most recent version, the YFAS 2.0, integrates DSM-5 criteria for substance use disorders, allowing its application to eating behaviors with addictive features (Gearhardt et al., 2016). During its development, the instrument underwent confirmatory factor analysis, which supported a unidimensional structure with diagnostic criteria loading onto a single latent factor and demonstrating high internal consistency (KR-20 = 0.92). This version has demonstrated robust psychometric properties across diverse populations, including healthy adults, patients with obesity, and individuals with eating disorders (Jahrami et al., 2025; Li et al., 2024).

The YFAS 2.0 has also proven useful in the bariatric surgery setting, serving as a valid measure for assessing the severity of food addiction symptoms and predicting postoperative outcomes (Koball et al., 2021). Moreover, its application has expanded to neurobiological research, behavioral interventions, and evaluations of therapeutic efficacy. Unlike other instruments, such as the Food Cravings Questionnaire (FCQ) or the Eating Disorder Examination Questionnaire (EDE-Q), the YFAS 2.0 provides a specific approach to dysfunctional eating behaviors within an addiction-based diagnostic framework, underscoring its clinical and operational relevance (López-Moreno et al., 2024; Meule et al., 2014). Furthermore, its high internal consistency and discriminative capacity have been consistently reported (Jahrami et al., 2025; Li et al., 2024; López-Moreno et al., 2024; Meule et al., 2014).

The YFAS 2.0 has been adapted into multiple languages, including Chinese, Hungarian, Spanish (Chile), Malay, and Danish, with validation studies reporting adequate psychometric performance across diverse populations (Chen et al., 2022; Tsegaye et al., 2025; Díaz-Torrente et al., 2024; Swarna Nantha et al., 2020; Horsager et al., 2020). Internal consistency values in these studies typically range from 0.85 to 0.97. Although the instrument was developed in the United States, international adaptations have supported the conceptual applicability of the construct across settings. Its widespread adoption in both clinical and research contexts has contributed to a growing body of cross-cultural evidence supporting the operationalization of food addiction as a measurable construct.

Despite its international dissemination, no formal psychometric validation of the YFAS 2.0 has been conducted in Mexican adults. Considering the significant epidemiological burden of obesity, T2DM, and other noncommunicable diseases in Mexico, as well as the urgent need for culturally adapted tools, this study aimed to perform the cross-cultural adaptation of the YFAS 2.0 and evaluate its psychometric properties in a sample of Mexican adults.

## 2. Materials and Methods

### 2.1. Study Design and Participants

A cross-sectional study was conducted to perform the cross-cultural adaptation and psychometric validation of the YFAS 2.0. Data were collected between March 2023 and February 2024 at a general zone hospital with family medicine services, part of a large metropolitan healthcare system in Mexico. This setting was selected due to its high outpatient volume and the sociodemographic diversity of the adult population it serves.

Adults aged over 20 years were recruited through non-probability sampling. This age threshold was selected to ensure consistency with national health surveys in Mexico, which define adults as individuals aged 20 years and older (Vargas et al., 2023). Participants were invited to take part voluntarily during their visits to the hospital, through direct invitations delivered by trained staff in waiting rooms and outpatient areas. Inclusion criteria comprised being 20 years of age or older, attending the hospital during the study period, and having the cognitive and legal capacity to provide informed consent. Exclusion criteria included a prior diagnosis of psychiatric or neurological disorders, pregnancy, hypothyroidism, and chronic steroid treatment, as these factors could interfere with behavioral assessment. Eligible participants completed an electronic questionnaire on digital tablets.

Sociodemographic data (age, sex, education level, marital status, and occupation), personal medical history, and current health status were collected. Age was treated as a continuous variable. Sex (male or female), education level (none, primary [i.e., elementary school], secondary [i.e., middle and high school], high school/technical, bachelor’s degree, or postgraduate), marital status (married, cohabiting, divorced, single, or widowed), and occupation (unemployed, student, homemaker, skilled worker, employee, professional, or retired) were treated as categorical variables.

### 2.2. Instrument

The YFAS 2.0 is a 35-item self-administered scale developed to assess addictive eating behaviors based on the 11 diagnostic criteria for substance use disorders outlined in the DSM-5. It evaluates symptoms such as tolerance, withdrawal, persistent desire, excessive intake, continued use despite adverse consequences, and impairment in social or personal functioning (Gearhardt et al., 2016).

The scale yields a continuous symptom score (0 to 11). It categorizes individuals according to diagnostic levels (mild, moderate, or severe) based on the number of criteria met and the presence of functional impairment.

In this study, the factorial structure of the YFAS 2.0 was also examined as part of the psychometric validation in the Mexican population.

### 2.3. Cross-Cultural Adaptation

The linguistic and cultural adaptation of the YFAS 2.0 followed international guidelines for cross-cultural adaptation of instruments (Beaton et al., 2000). The scale was independently translated by two bilingual translators. Both versions were reconciled and back-translated into English by two additional translators blinded to the original instrument. Back-translations were compared with the original version to ensure semantic and conceptual equivalence.

An expert panel assessed the clarity, cultural relevance, and content validity of each item. Minor wording adjustments were made based on their feedback. A pilot test was conducted with 25 individuals from the target population to assess item comprehension, clarity, and cultural relevance, following methodological recommendations for preliminary scale testing (Johanson & Brooks, 2010). Participants completed the full version of the translated instrument and provided structured feedback. Minor modifications were made based on their comments, primarily in the introductory section of the translated instrument describing food categories. These changes involved adapting examples of foods and beverages to reflect the linguistic and cultural context of the Mexican population, while preserving the conceptual meaning of each item. In addition, small wording adjustments were made in a limited number of items (1, 6, 14, 20, and 28) to improve clarity without altering the original construct. No items were removed or added. All participants reported that the content was understandable and appropriate. These modifications were incorporated prior to the final administration of the scale. The Spanish-language version of the final adapted YFAS 2.0 used in this study is available in the Supplementary Materials.

### 2.4. Statistical Analysis

Exploratory and confirmatory factor analyses were conducted on two independent subsamples drawn from the total sample (N = 500). For the exploratory analysis, maximum likelihood extraction with Varimax rotation was applied. This orthogonal rotation method was selected to clarify the underlying factor structure without assuming correlation between factors, allowing us to evaluate whether the scale supported a unidimensional or multifactorial solution. The explained variance for each factor and the eigenvalues were obtained, as well as the scree plot. Adequacy criteria included a Kaiser–Meyer–Olkin (KMO) index ≥0.80 and a significant Bartlett’s test of sphericity (*p* < 0.05).

Confirmatory factor analysis was conducted using structural equation modeling. Acceptable fit indices included a Comparative Fit Index (CFI) ≥ 0.90, Tucker–Lewis Index (TLI) ≥ 0.90, and Root Mean Square Error of Approximation (RMSEA) ≤ 0.08. The Root Mean Square Residual (RMR) was approximately 0, the Normed Fit Index (NFI) ranged between 0.90 and 1.00, and total omega and hierarchical omega coefficients were ≥0.70. Internal consistency was estimated using Cronbach’s alpha coefficient, with ≥0.70 considered acceptable.

Statistical analyses were performed using SPSS version 30 and JAMOVI 2.6.44.

### 2.5. Ethical Considerations

This study was approved by the Research Ethics Committee of the Mexican Social Security Institute (IMSS) (R-2023-3605-094) and the Ethics Committee of the Faculty of Medicine at the National Autonomous University of Mexico (UNAM) (FMED/CEI/MHU/037/2020). All participants provided written informed consent prior to enrollment. Participation was voluntary, and data were treated confidentially and anonymized in accordance with the principles of the Declaration of Helsinki and applicable national regulations (López-Pacheco et al., 2016; World Medical Association, 2024).

## 3. Results

### 3.1. Study Population Characteristics

The sample comprised 500 participants, with a mean age of 46.2 years (±17.6; range: 20–87 years). Women accounted for 66.0% (*n* = 330) of the sample. The most frequent educational levels were higher education (37.2%) and upper secondary education (31.0%). Regarding employment, 41.6% were classified as non-professional employees and 16.6% reported being homemakers. Concerning marital status, 39.4% of participants were single and 30.4% were married (Table 1).

The mean body mass index (BMI) was 27.8 ± 5.2 (range: 17.8–52.7). Women had a mean BMI of 28.0 ± 5.4 and men 27.3 ± 4.7. Overall, 31.6% had normal weight, 40.4% were overweight, and 27.4% were classified as obese. Among obese participants, 19.6% had class 1 obesity, 5.8% class 2, and 2% class 3. Mean waist circumference was 91.24 ± 14.10 cm in men and 90.24 ± 15.17 cm in women.

### 3.2. Factorial Model

An exploratory factor analysis (EFA) was conducted in a sample of 200 participants using the maximum likelihood extraction method with Varimax rotation. The adequacy of the correlation matrix was confirmed by a Kaiser–Meyer–Olkin (KMO) index of 0.815 and a significant Bartlett’s test of sphericity (χ^2^ = 4367.88; df = 595; *p* < 0.001).

The first factor explained 21.3% of the total variance, with a ratio between the first and second eigenvalues greater than 3 (Table 2). This pattern justified a focused analysis of the five items with the highest loadings (items 4, 25, 28, 31, and 32), which revealed a unidimensional structure explaining 40.33% of the variance (Table 3).

**Figure 1:**
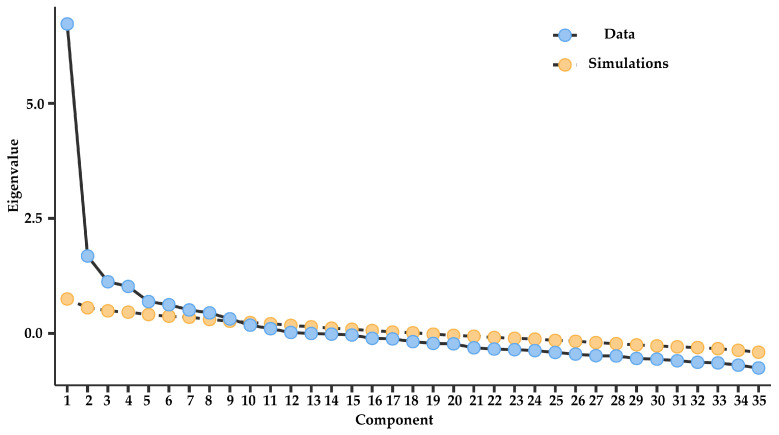
Scree plot from the exploratory factor analysis of the YFAS 2.0 (unidimensional model).

The scree plot showed a sharp decline in eigenvalues after the first factor, with the slope leveling off from the second component onward. This pattern is compatible with a unidimensional structure, as the largest proportion of explained variance is concentrated in the first factor, while subsequent factors account for smaller portions (Figure 1).

Based on the unidimensional structure identified in the exploratory factor analysis and the concentration of variance in the first factor, a hierarchical factor analysis was subsequently conducted in a subsample of 326 participants. All items showed substantial loadings on the general factor, with items 31 and 32 presenting the highest loadings (0.958 and 0.956, respectively), supporting the presence of a dominant unidimensional structure (Table 4).

Global fit indices and reliability coefficients corresponding to the hierarchical model were estimated. Table 5 presents the resulting measures, including residual-based indicators (RMR and RMSEA), incremental fit indices (CFI, TLI, and NFI), and internal consistency estimates (total and hierarchical omegas).

### 3.3. Internal Consistency

Internal consistency of the YFAS 2.0 scale was assessed in the study population using Cronbach’s alpha. A high level of consistency was identified, with an alpha coefficient of 0.88.

## 4. Discussion

This study provides evidence for the cross-cultural adaptation and psychometric validation of the Yale Food Addiction Scale 2.0 (YFAS 2.0) in a sample of Mexican adults. In a country where obesity and metabolic disorders remain highly prevalent, having reliable instruments to assess constructs such as food addiction is essential for developing effective public health and clinical strategies. To assess the latent structure of the instrument in this context, a sequence of exploratory and hierarchical analyses was conducted.

An initial exploratory analysis yielded a two-factor solution; however, the second factor was poorly defined and driven primarily by two items with exceptionally high loadings, while most other items contributed marginally. A hierarchical bifactor model was also tested, but no interpretable or stable subdimensions emerged. The findings instead supported a strong general factor structure, aligning with a unidimensional hierarchical model in which a single latent construct accounted for most of the shared variance.

The results support the structural validity of the scale in this population. Exploratory factor analysis identified a unidimensional structure, with the first factor explaining 21.3% of the total variance—above the threshold proposed for relevant latent dimensions. In addition (Reckase, 1979), the ratio between the first and second eigenvalues was greater than 3, a criterion considered indicative of unidimensionality (Hattie, 1985). These findings were further confirmed through a hierarchical factor model, in which all items loaded substantially onto the general factor, particularly items 31 and 32, with loadings above 0.95.

The retention of a unidimensional structure in this study is consistent with the original validation of the YFAS 2.0, where Gearhardt et al. (2016) compared a single-factor model with a two-factor solution based on DSM-5 abuse and dependence domains. The two-factor model showed no meaningful improvement in model fit and revealed high interfactor collinearity (r = 0.90), supporting the use of a single latent construct. This structure reflects the empirical convergence of the 11 diagnostic criteria into a cohesive pattern of compulsive eating, rather than distinct symptom domains. Although dimensions such as craving, withdrawal, or social impairment may vary in clinical presentation, their factorial overlap supports a shared underlying mechanism—consistent with transdiagnostic models of addiction. This configuration does not preclude item-level analysis or clinical profiling, but it enhances the reliability and coherence of global scores, which are often used in public health screening, prevalence estimates, and large-scale risk stratification. In clinical settings, a unitary score simplifies decision-making processes and improves comparability across populations, while still allowing for qualitative exploration of individual symptom patterns when needed. Thus, the chosen structure balances psychometric parsimony with diagnostic utility, reinforcing its suitability for both research and practice.

This pattern of results aligns with findings from other cultural validations of the YFAS 2.0 that also retained a unidimensional structure using ordinal item formats (Brunault et al., 2020; Chen et al., 2022; Díaz-Torrente et al., 2024). These findings support the conceptual stability of the YFAS 2.0 across different populations and methodological approaches.

Beyond structural convergence, item-level comparisons also reveal relevant patterns. In the present study, five items showed very high loadings (>0.85), while most ranged from 0.30 to 0.75. This variability is consistent with the clinical nature of the scale, which includes both frequent and infrequent behaviors. In contrast, the original validation reported uniformly high loadings (≥0.77; Gearhardt et al., 2016), the Spanish version found loadings above 0.80 for nearly all items (Granero et al., 2018), and the Chilean adaptation reported loadings >0.69 (Díaz-Torrente et al., 2024). Despite these differences, all studies—including the present one—support a unidimensional latent structure, with a dominant general factor and excellent model fit.

Similar findings have been reported in other cultural contexts. In Chile, a unidimensional model with satisfactory fit indices was confirmed, while a two-factor alternative aligned with DSM-5 criteria was discarded due to high collinearity (Díaz-Torrente et al., 2024). In Taiwan, a unidimensional structure was also validated for both versions of the scale, and a second-order hierarchical model was explored, suggesting the presence of a dominant general factor (Chen et al., 2022). These observations indicate that hierarchical and unidimensional models are methodologically appropriate when validating the YFAS 2.0 in heterogeneous populations.

The internal consistency of the scale was high (Cronbach’s α = 0.88), in line with results from a global meta-analysis by Jahrami et al., which reported an average internal consistency of α = 0.85 across more than 50 studies, along with a mean test–retest reliability of ICC = 0.77. While these values support the scale’s reliability across settings, the meta-analysis also emphasized the lack of longitudinal evidence regarding temporal stability. Future research should address this gap, particularly in clinical populations, if the YFAS 2.0 is to be used as a diagnostic or monitoring tool for food addiction-related interventions.

A substantial proportion of participants were overweight (40.4%) or obese (27.4%), consistent with recent epidemiological data on Mexican adults (Barquera et al., 2024; Escamilla-Nuñez et al., 2023; Rojas-Martínez et al., 2024; Institute for Health Metrics and Evaluation, 2025). This reinforces the scale’s relevance for clinical and public health use, particularly as a screening tool for dysfunctional eating behaviors associated with metabolic comorbidities.

From a neurobiological perspective, food addiction has been linked to functional alterations in key brain reward regions, including the nucleus accumbens, prefrontal cortex, and insula—structures also implicated in substance use disorders (Fletcher & Kenny, 2018; Ravichandran et al., 2024). Based on DSM-5 diagnostic criteria, the YFAS 2.0 offers a standardized framework that facilitates clinical application and supports rigorous cross-cultural comparisons.

Additionally, food addiction has been linked to social determinants such as chronic stress, food insecurity, and the environmental availability of ultra-processed foods (Halbeisen et al., 2025). A psychometrically sound and culturally adapted tool like the YFAS 2.0 makes it possible to assess not only individual consumption patterns but also the influence of social and environmental factors. In settings dominated by readily available and heavily marketed ultra-processed foods, these structural factors may promote compulsive intake even in individuals without an apparent psychological predisposition. Integrating the YFAS 2.0 in studies addressing these variables may expand the ecological understanding of food addiction and inform more comprehensive interventions (Belfiore et al., 2024; Gearhardt, 2021; Minhas et al., 2021; Varnado et al., 2024).

Although food addiction has not yet been officially recognized as a clinical diagnosis in the DSM-5 or ICD-11, accumulating empirical evidence supports its consideration as a valid behavioral phenotype within the spectrum of eating disorders. The development of standardized instruments such as the YFAS 2.0 has helped define its clinical dimensions and generate quantifiable evidence that may, in the future, support its formal inclusion in international diagnostic systems (LaFata et al., 2024; Oliveira et al., 2021; Tsegaye et al., 2025).

Finally, cross-cultural adaptation of assessment tools remains essential for developing psychometric evidence, particularly in linguistically and culturally diverse settings (Tsegaye et al., 2025). This study reinforces the importance of locally validating internationally recognized instruments to ensure conceptual and operational relevance. The present version of the YFAS 2.0 offers a reliable methodological resource for both clinical research and the development of culturally relevant public health strategies.

While this study yielded results consistent with the international literature, it has limitations. The non-probabilistic sample may restrict the generalizability of findings to other demographic groups. In particular, participants were recruited from a single urban hospital in Mexico City, limiting geographic and cultural representativeness. Future studies should consider stratified or multicentric sampling designs to capture greater regional and sociocultural diversity across Mexico. Additionally, convergent and discriminant validity analyses were not conducted. Future research should incorporate complementary scales, such as the Eating Disorder Examination Questionnaire and the Food Cravings Questionnaire, and compare the YFAS 2.0 with clinical diagnostic criteria (Ahuja et al., 2023; Bang et al., 2023; Gearhardt et al., 2016). Furthermore, diagnostic sensitivity and specificity should be examined against other eating disorders, such as binge eating disorder and bulimia nervosa, to establish the scale’s differential utility in real-world clinical settings. Finally, as with any self-report instrument, responses may be influenced by social desirability bias, potentially leading to underreporting of problematic eating behaviors.

Despite these limitations, the YFAS 2.0 demonstrated adequate psychometric properties in this population and represents a useful tool for identifying addictive eating patterns. Its application could strengthen clinical and public health strategies aimed at managing overweight, obesity, and other metabolic disorders. Implementation studies in primary care and at-risk populations will help expand its operational validation and contribution to evidence-based behavioral interventions.

## 5. Conclusions

This study supports the validity and reliability of the YFAS 2.0 in a sample of Mexican adults. The results confirmed a unidimensional hierarchical structure with acceptable fit indices and internal consistency. While some variability in item loadings was observed, the overall model aligns with findings from previous validations in other populations.

The adapted version provides a standardized instrument suitable for use in clinical and public health research. Its integration into primary care and population-based studies may support the early identification of dysfunctional eating patterns and inform strategies for managing obesity and related conditions. Further research in clinical populations and diverse demographic groups is recommended to expand its applicability.

## Figures and Tables

**Table 1:** Study population characteristics.

Characteristic	Men Frequency (%)	Women Frequency (%)	*p*-Value
**Sex**	170 (34.0)	330 (66.0)	<0.0001
**Age (years)**			0.82
20–29	36 (21.18)	70 (21.21)	
30–39	30 (17.65)	71 (21.52)	
40–49	21 (12.35)	40 (12.12)	
50–59	18 (10.59)	59 (17.88)	
60–69	28 (16.47)	59 (17.88)	
70 and older	27 (15.88)	31 (9.39)	
**Educational level**			0.44
None	3 (0.91)	3 (0.91)	
Primary (i.e., elementary school)	10 (5.55)	36 (10.91)	
Secondary (i.e., middle and high school)	20 (11.76)	62 (18.79)	
High school/Technical	64 (37.65)	91 (27.58)	
Bachelor’s degree	67 (39.41)	119 (36.06)	
Postgraduate	6 (3.53)	19 (5.76)	
**Occupation**			0.01
Unemployed	2 (1.18)	1 (0.30)	
Student	17 (10.0)	35 (10.61)	
Homemaker	0	83 (25.15)	
Skilled worker	17 (10.0)	10 (3.03)	
Employee	85 (50.0)	123 (37.37)	
Professional	13 (7.65)	46 (13.94)	
Retired	36 (21.18)	32 (9.70)	
**Marital status**			0.07
Married	61 (35.88)	91 (27.58)	
Cohabiting	27 (15.88)	53 (16.06)	
Divorced	4 (2.35)	26 (7.88)	
Single	70 (41.18)	127 (38.48)	
Widowed	8 (4.71)	33 (10.0)	

*p*-values calculated using χ^2^ test.

**Table 2:** Total variance explained for the YFAS 2.0 instrument (exploratory factor analysis).

	Initial Eigenvalues			Extraction Sums of Squared Loadings		
Factors	Total	% of Variance	Cumulative %	Total	% of Variance	Cumulative %
1	7.45	21.302	21.30	5.096	14.56	14.56
2	2.307	6.592	27.89	3.513	10.03	24.37
3	1.983	5.666	33.55			

Extraction method: Maximum likelihood.

**Table 3:** Total variance explained for the YFAS 2.0 instrument (Unidimensional Model).

	Initial Eigenvalues			Extraction Sums of Squared Loadings		
Factors	Total	% of Variance	Cumulative %	Total	% of Variance	Cumulative %
1	2.017	40.33	40.33	1.51	30.22	1.48
2	1.134	22.685	63.01	0.569	11.37	41.601
3	2.16	6.16	45.46			

Extraction method: Maximum likelihood. Goodness-of-fit test: χ^2^ = 5694.60 (*p* < 0.001).

**Table 4:** Factor loadings of the YFAS 2.0 items.

Item	Dominant Factor *	Remaining Scale Items *
Item 31	0.958	
Item 32	0.956	
Item 25	0.729	
Item 4	0.558	
Item 28	0.484	
Item 13		0.807
Item 12		0.753
Item 11		0.551
Item 14		0.390
Item 5		0.581
Item 1		0.535
Item 2		0.472
Item 23		0.438
Item 22		0.432
Item 3		0.418
Item 16		0.396
Item 15		0.336
Item 6		0.320
Item 30		0.858
Item 29		0.665
Item 7		0.388
Item 26		0.379
Item 34		0.865
Item 33		0.788
Item 9		0.862
Item 35		0.626
Item 8		0.379
Item 17		0.638
Item 19		0.525
Item 27		0.352
Item 21		0.778
Item 20		0.513
Item 10		0.303
Item 24		0.611
Item 18		0.553

* Hierarchical factor model.

**Table 5:** Hierarchical reliability and fit indices (YFAS 2.0).

Index	Value
Root Mean Square Residual (RMR)	0.004
Root Mean Square Error of Approximation (RMSEA)	0.001
Comparative Fit Index (CFI)	0.99
Tucker–Lewis Index (TLI)	0.98
Normed Fit Index (NFI)	0.98
Total omega (ωt)	0.87
Hierarchical omega (ωh)	0.89

## Data Availability

The data presented in this study are not publicly available due to ethical restrictions and institutional regulations regarding the use of patient medical information. Access to the dataset may be considered upon reasonable request and with permission from the Instituto Mexicano del Seguro Social (IMSS).

## Supplementary Materials

The following supporting information can be downloaded at https://www.mdpi.com/article/10.3390/bs15081023/s1, Spanish-language version of the final adapted YFAS 2.0 for the Mexican adult population.
