# Supplementary material for: Cross-Cultural Adaptation and Psychometric Validation of the YFAS 2.0 for Assessing Food Addiction in the Mexican Adult Population
Source: Behav Sci (Basel). 2025 Jul 28;15(8):1023. doi: 10.3390/bs15081023 (PMC12382649; doi:10.3390/bs15081023)
Supplement: Supplementary file 1 [file behavsci-15-01023-s001.zip › behavsci-3706507-supplementary.pdf]

Supplementary Material

Spanish-Language Version of the Final Adapted YFAS 2.0 for the Mexican Adult Population

YFAS 2.0 (Escala de Adicción a los Alimentos de Yale 2.0)

Las siguientes preguntas se refieren a su alimentación durante los últimos 12 meses. Algunas personas tienen dificultades para controlar cuánto comen de ciertos alimentos, especialmente aquellos con alto contenido de azúcar, grasa o sal. Entre estos alimentos se incluyen, por ejemplo:

- Dulces como helados, chocolates, galletas, pasteles, pan de dulce o gomitas.
- Comidas con harinas, como pan blanco, tortillas, tamales, pasta, arroz o cereales de caja como Corn Flakes.
- Botanas saladas, como papas fritas, chicharrones, palomitas, frituras de maíz condimentadas o enchiladas (como churritos o totopos), galletas saladas y cacahuates.
- Comidas muy grasosas, como tacos, tortas, quesadillas, carnitas, hamburguesas, pizzas o alimentos fritos en aceite.
- Bebidas azucaradas como refrescos, aguas frescas de sabor con azúcar, jugos de cajita o botella y bebidas deportivas o energéticas dulces (con azúcar).

Cuando las siguientes preguntas se refieren a "algunos alimentos", piense en cualquier alimento o bebida similar a los que figuran en los grupos de alimentos o bebidas mencionados anteriormente o cualquier otro alimento con el que haya tenido dificultades durante el año pasado.

En los últimos 12 meses	Nunca	Menos de una vez al mes	Una vez al mes	De 2 a 3 veces al mes	Una vez a la semana	De 2 a 3 veces por semana	De 4 a 6 veces por semana	Diario
1. Cuando como algunos alimentos, me doy cuenta de que he comido mucho más de lo planeado.	0	1	2	3	4	5	6	7
2. Continúo comiendo algunos alimentos, aunque ya no tenga hambre.	0	1	2	3	4	5	6	7
3. He comido hasta el punto de sentirme mal por tanto comer.	0	1	2	3	4	5	6	7
4. Me ha preocupado mucho reducir el consumo de algunos tipos de alimentos, pero los como de todos modos.	0	1	2	3	4	5	6	7
5. He pasado mucho tiempo sintiéndome lento o cansado por comer en exceso.	0	1	2	3	4	5	6	7
6. He pasado mucho tiempo comiendo algunos alimentos durante todo el día.	0	1	2	3	4	5	6	7
7. Cuando algunos alimentos no han estado disponibles, he hecho todo lo posible por conseguirlos. Por ejemplo, he ido a la tienda a comprar esos alimentos o comida chatarra, incluso si tenía otros disponibles en casa.	0	1	2	3	4	5	6	7
8. He comido algunos alimentos con tanta frecuencia o en cantidades tan grandes que he dejado de hacer otras cosas importantes. Estas cosas pueden haber sido trabajar o pasar tiempo con familiares o amigos	0	1	2	3	4	5	6	7
9. He tenido problemas con mi familia o amigos debido a lo mucho que como.	0	1	2	3	4	5	6	7
10. He evitado el trabajo, la escuela o las actividades sociales por miedo de comer en exceso allí.	0	1	2	3	4	5	6	7
11. Cuando he reducido o dejado de comer algunos alimentos, me he sentido irritable, nervioso o triste.	0	1	2	3	4	5	6	7

12. Si me sintiera mal porque no he comido algunos alimentos, los comería para sentirme mejor.	0	1	2	3	4	5	6	7
13. Si tuviera problemas emocionales porque no he comido algunos alimentos, los comería para sentirme mejor.	0	1	2	3	4	5	6	7
14. Cuando he reducido o dejado de consumir ciertos alimentos, he experimentado síntomas físicos (por ejemplo, dolor de cabeza, fatiga u otros).	0	1	2	3	4	5	6	7
15. Cuando he reducido o dejado de comer algunos alimentos, he tenido fuertes antojos por ellos.	0	1	2	3	4	5	6	7
16. Mi comportamiento alimenticio me ha causado mucha angustia.	0	1	2	3	4	5	6	7
17. He tenido problemas importantes en mi vida por la comida y la alimentación. Estos me ocasionaron problemas con mi rutina diaria, trabajo, escuela, amigos, familia o salud.	0	1	2	3	4	5	6	7
18. Me he sentido tan mal por comer en exceso que no hice otras cosas importantes, como trabajar o pasar tiempo con familiares o amigos.	0	1	2	3	4	5	6	7
19. Comer en exceso me ha impedido cuidar a mi familia o hacer las tareas del hogar.	0	1	2	3	4	5	6	7
20. He evitado el trabajo, la escuela o las relaciones sociales porque no podía comer algunos alimentos allí.	0	1	2	3	4	5	6	7
21. He evitado situaciones sociales porque la gente me juzgaría por cuánto podía comer.	0	1	2	3	4	5	6	7
22. He seguido comiendo de la misma manera a pesar de que mi alimentación me ha causado problemas emocionales.	0	1	2	3	4	5	6	7
23. He seguido comiendo de la misma manera a pesar de que mi alimentación me causa problemas físicos.	0	1	2	3	4	5	6	7
24. Comer la misma cantidad de comida no me da tanto placer como solía hacerlo.	0	1	2	3	4	5	6	7
25. He tenido muchas ganas de reducir o dejar de comer algunos tipos de alimentos, pero no puedo.	0	1	2	3	4	5	6	7
26. He necesitado comer más y más para sentirme satisfecho, comer en exceso ha reducido en mí emociones negativas como la tristeza o ha incrementado mi placer.	0	1	2	3	4	5	6	7
27. No me ha ido bien en el trabajo o en la escuela porque comía demasiado.	0	1	2	3	4	5	6	7
28. He seguido comiendo algunos alimentos aun sabiendo que es físicamente peligroso. Por ejemplo, seguía comiendo dulces aunque tenía diabetes, o seguí comiendo alimentos grasos a pesar de tener una enfermedad cardíaca.	0	1	2	3	4	5	6	7
29. He tenido tantas ganas de comer algunos alimentos que no puedo pensar en otra cosa.	0	1	2	3	4	5	6	7
30. He tenido antojos tan intensos por algunos alimentos que sentí que tenía que comerlos de inmediato.	0	1	2	3	4	5	6	7
31. He tratado de reducir o no comer algunos tipos de alimentos, pero no he tenido éxito.	0	1	2	3	4	5	6	7
32. He tratado y no pude reducir o dejar de comer algunos alimentos.	0	1	2	3	4	5	6	7
33. He estado tan distraído al comer que pude haberme lastimado (p. Ej., Al conducir un automóvil, cruzar la calle, operar maquinaria).	0	1	2	3	4	5	6	7
34. He estado tan distraído al pensar en la comida que podría haberme lastimado (p. Ej., Al conducir un automóvil, cruzar la calle, operar maquinaria).	0	1	2	3	4	5	6	7
35. Mis amigos o familiares han estado preocupados porque como en exceso.	0	1	2	3	4	5	6	7
